# Comprehensive analysis of non-coding RNA-mediated endothelial cell-specific regulatory circuits in coronary artery disease risk

**DOI:** 10.3389/fgene.2025.1559798

**Published:** 2025-02-21

**Authors:** Boshui Huang, Zhijie Lai, Xiaoyu Wang, Qinhao Zhang, Tingting Hu, Fulong Yu, Shuxian Zhou, Yan Zhang, Juan Meng

**Affiliations:** ^1^ Division of Cardiology, Sun Yat-Sen Memorial Hospital, Sun Yat-Sen University, Guangzhou, China; ^2^ Division of Cardiology, Puning People’s Hospital, Puning, China; ^3^ Medical Research Institute, Guangdong Provincial People’s Hospital (Guangdong Academy of Medical Sciences), Southern Medical University, Guangzhou, China; ^4^ GMU-GIBH Joint School of Life Sciences, Guangzhou Medical University, Guangzhou, China; ^5^ Department of Geriatric Medicine, National Clinical Research Center for Infectious Diseases, The Third People’s Hospital of Shenzhen, Shenzhen, China

**Keywords:** coronary artery disease (CAD), circular RNA (circRNA), GWAS, endothelial cell (EC), genetic variants

## Abstract

Coronary artery disease (CAD) remains the leading cause of mortality worldwide, driven by both lifestyle factors and genetic predisposition. Large-scale population genetic studies have greatly enhanced our understanding of the genetic underpinnings of CAD and facilitated the discovery of disease-associated genes. Noncoding RNAs, such as circular RNAs (circRNAs) and microRNAs (miRNAs), play crucial roles in the regulation of these genes. However, the impact of CAD-associated genetic variants on noncoding RNAs and their regulatory gene networks remain largely unexplored. In this study, we systematically identified the targets of both noncoding and coding genes influenced by CAD-associated variants. We constructed a CAD risk gene network, encompassing circRNAs, miRNA and genes, based on the concept of competing endogenous RNA regulation. Additionally, we focused on the endothelial cell (EC)-specific gene regulatory network to prioritize disease-associated circRNAs. Notably, we identified two CAD-associated variants that may disrupt circZNF609 and circABCC1, potentially altering their function as miRNA sponges and impacting EC-specific gene regulation, ultimately contributing to disease risk. Our findings link CAD genetic predisposition to noncoding RNA-mediated gene regulatory mechanisms in specific cell types, providing a valuable resource for novel target identification and advancing precision medicine in CAD.

## Introduction

Coronary artery disease (CAD) is the most common type of heart disease and a leading cause of morbidity and mortality worldwide (LaRocca et al., 2017; Campbell et al., 2021). The complexity of CAD arises from the involvement of various cell types, including endothelial cells (ECs), smooth muscle cells, fibroblasts, and immune cells, which can undergo phenotypic changes (Zhu et al., 2024). Among these, ECs play a critical role as the primary site where atherosclerosis develops, leading to CAD (Schnitzler et al., 2024). Understanding the regulatory mechanisms that malfunction in ECs is crucial for developing effective treatments for CAD.

Circular RNAs (circRNAs) are a class of non-coding RNAs characterized by their high stability and tissue or cell type-specific expression patterns (Kristensen et al., 2019; Yu et al., 2018; Tan et al., 2017). Circular RNAs (circRNAs) can modulate gene expression through various mechanisms, including acting as microRNA (miRNA) sponges. By sequestering miRNAs, circRNAs prevent them from binding to their target messenger RNAs (mRNAs), thereby regulating gene expression (Tay et al., 2014; Zhang et al., 2018; Glaser et al., 2023). Increasing evidence suggests that non-coding RNAs, including circRNAs and miRNAs, are key players in numerous cellular processes, and their dysregulation is linked to the pathogenesis of several cardiovascular diseases, including CAD (Zhang et al., 2020a; Zhang et al., 2015; Zhang et al., 2020b). Consequently, these molecules are being investigated as promising therapeutic targets for CAD. However, a comprehensive understanding of the cell type-specific regulation mediated by non-coding RNAs in CAD is still lacking.

Genome-wide association studies (GWAS) have been instrumental in identifying thousands of risk loci associated with CAD, providing new insights into the disease’s etiology, and offering potential targets for drug development (Yu et al., 2022). Recent studies have explored the genetic regulation of circRNA expression and its potential link to cardiovascular diseases. For example, research on human aortic smooth muscle cells has identified circRNA quantitative trait loci (circQTLs) that colocalize with GWAS loci associated with CAD. Notably, some genetic variants specifically affect circRNA expression without altering the expression of their linear mRNA counterparts, suggesting that circRNAs may contribute uniquely to the genetic architecture of CAD. While some CAD-associated GWAS loci have been linked to circRNAs (Aherrahrou et al., 2023), most of these loci are non-coding and their functional roles remain largely unexplored. The impact of CAD-associated genetic variants on gene regulatory networks, including both coding genes and non-coding RNAs such as circRNAs within endothelial cells (ECs), remains unclear.

In this study, we aimed to elucidate the complex molecular mechanisms underlying CAD by focusing on the roles of circRNAs in ECs. Through a comprehensive analysis, we identified the CAD-associated circRNAs, miRNAs, and gene sets, constructing a circRNA competitive regulatory network. This network uncovered intricate interactions between genetic variants and circRNAs, with a particular emphasis on circZNF609 and circABCC1. Our findings indicate that specific CAD-associated genetic variants may alter the function of circZNF609 and circABCC1, thereby influencing gene regulation in ECs. These circRNAs likely act as miRNA sponges, sequestering miRNAs and thus modulating the expression of downstream genes. The dysregulation of these gene programs may contribute to CAD pathogenesis by impairing endothelial cell function and promoting atherosclerosis. This study provides new insights into how genetic predisposition influences CAD risk through non-coding RNA-mediated regulatory mechanisms.

## Materials and methods

### Identification of circRNA-mRNA competitive interactions

We sourced human circRNA-miRNA and miRNA-mRNA interactions from RAID v2.0 (Yi et al., 2017), aggregating RNA-associated interactions from experimental data and computational predictions in public databases. We retained interactions with a confidence score exceeding 0.3. A circRNA-mRNA pair sharing at least three miRNAs was considered competitively regulated.

### GWAS variants for CAD

The GWAS variants associated with CAD were obtained from studies by Aragam et al. (2022) and van der Harst and Verweij (2018). For each GWAS signal, we defined a set of nearby circRNAs and genes, encompassing the two closest circRNAs and genes on either side and all within ±500 kb. This yielded a total of 1,942 candidate GWAS circRNAs and genes.

### Known CAD-associated circRNAs, miRNAs and genes

The CAD-associated circRNAs were sourced from circRNADisease v2.0 (Sun et al., 2024). Known CAD miRNAs were obtained from HMDD v4.0 (Cui et al., 2024) and miR2Disease (Jiang et al., 2009), while CAD-associated genes were sourced from OMIM (Amberger and Hamosh, 2017) and GeneCards (Stelzer et al., 2016). The summary of the CAD-associated circRNAs, miRNAs and genes can be found in the Supplementary Table S1.

### Expression data of circRNA and mRNA in CAD

The circRNA expression profile (GSE115733) from 24 CAD patients and seven healthy samples, and mRNA expression data (GSE23561) from six CAD patients and nine healthy samples, were retrieved from the GEO database. Samples were derived from peripheral blood, normalized, and log2 transformed. Differentially expressed circRNAs and genes were identified using adj.p < 0.05 and |fold change|>1.5. The DE circRNA and gene list can be seen in the Supplementary Table S2.

### Construction of CAD risk circRNA-gene network

The CAD-associated circRNAs/genes were mapped into the global circRNA-mRNA competitive network. We kept the circRNA-mRNA interactions with at least one GWAS or known or DE circRNAs/genes to construct the CAD risk circRNA network. Network visualization was conducted using Cytoscape (Majeed and Mukhtar, 2023) and GO functional enrichment analysis was performed using the R package clusterprofiler (Wu et al., 2021).

### EC-specific programs in CAD

To explore EC-specific dysfunction pathways in CAD, Jesse M. Engreitz lab used Perturb-seq and scRNA-seq data to systematically identify sets of genes that act together in biological pathways (Schnitzler et al., 2024). A total of 13 EC-specific programs were found. Each program had 300 genes.

## Results

### The global circRNA-miRNA-mRNA competitive triplets

**FIGURE 1:**
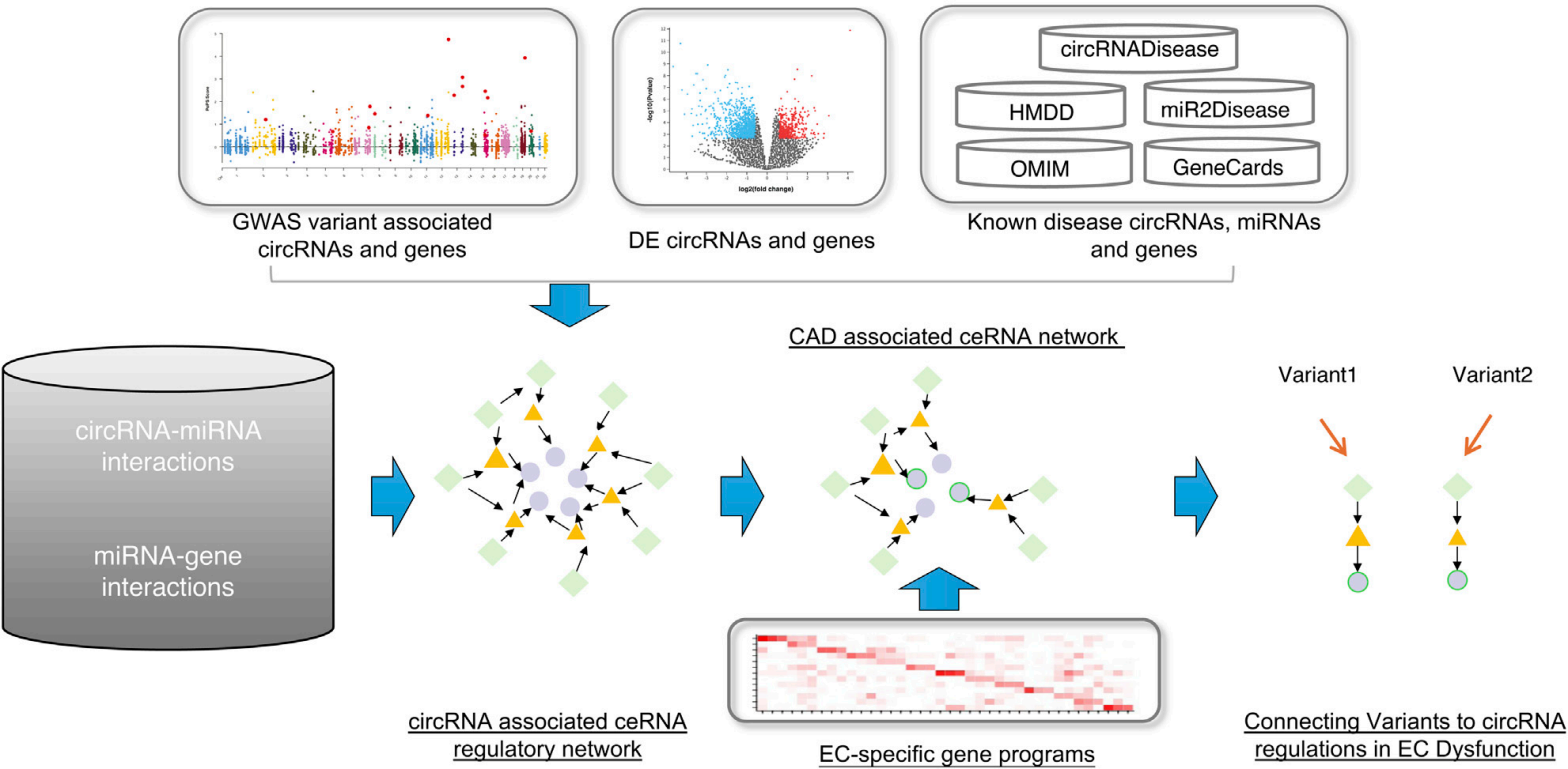
The workflow of this study. First, construct the global circRNA-associated ceRNA network. Second, collected the CAD-associated circRNAs, miRNAs and genes and then mapped them into the background network to identify the CAD-associated ceRNA network. At last, using the EC-specific gene programs, we connected variants to circRNA regulations in EC dysfunction. Green diamonds represent circRNAs, yellow triangles represent miRNAs, and light purple circles represent genes.

The workflow of our approach is illustrated in Figure 1. To identify global circRNA-miRNA-mRNA competitive triplets, we extracted high-confidence human circRNA-miRNA and miRNA-mRNA interactions from the RAID database. Triplets were defined by the presence of at least three shared miRNAs, resulting in the identification of 2,089,725 triplets, comprising 478 circRNAs, 279 miRNAs and 9,908 genes.

**FIGURE 2:**
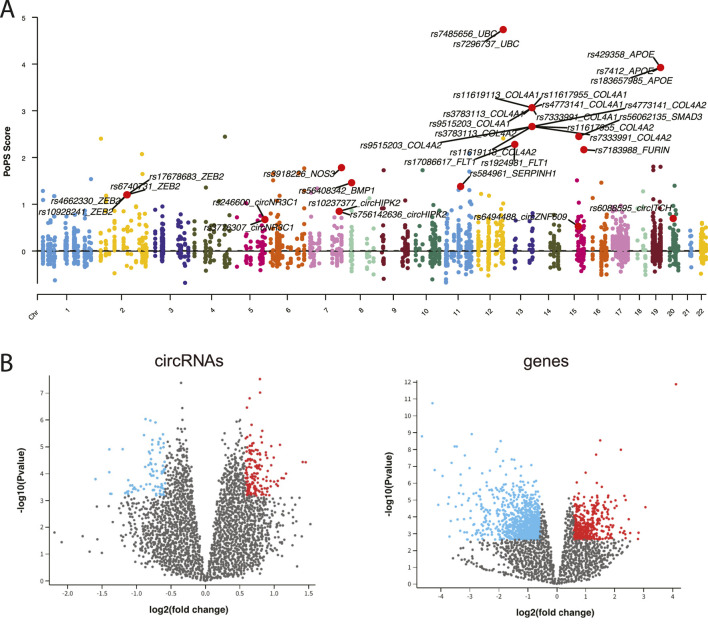
**(A)** Manhattan plot displaying CAD GWAS variants. **(B)** Volcano plot illustrating the differential expression analysis of circRNAs and genes.

To establish connections between circRNA regulations and CAD associations, we integrated comprehensive datasets of CAD-associated circRNAs, miRNAs and genes. From 307 CAD GWAS variants, we identified 90 nearby circRNAs and 2,720 nearby genes. Notably, circZNF609 and circHERPUD2, located near CAD disease variants, have been suggested as potential biomarkers for CAD (Liang et al., 2020; He et al., 2023). Similarly, genes near these variants, such as *MMP3*, *SMAD3*, *COL4A1*, and *COL4A2*, have known associations with CAD. Additionally, other genes like *UBC* and *APOE*, located near causal variants, may represent novel CAD biomarkers (Figure 2A).

Using expression data from peripheral blood mononuclear cells (PBMCs) of CAD patients and healthy controls, we identified 125 differentially expressed (DE) circRNAs and 1,245 DE genes (adj.p < 0.05 and |fold change|>1.5) (Figure 2B). Furthermore, we retrieved 29 known CAD circRNAs, 54 known CAD genes, and 179 known CAD miRNAs from manually curated disease databases. This comprehensive analysis yielded a total of 241 CAD-associated circRNAs, 179 CAD-associated miRNAs, and 3,872 CAD-associated genes (Supplementary Figure S1).

### The risk circRNA-mRNA competitive regulatory network in CAD

**FIGURE 3:**
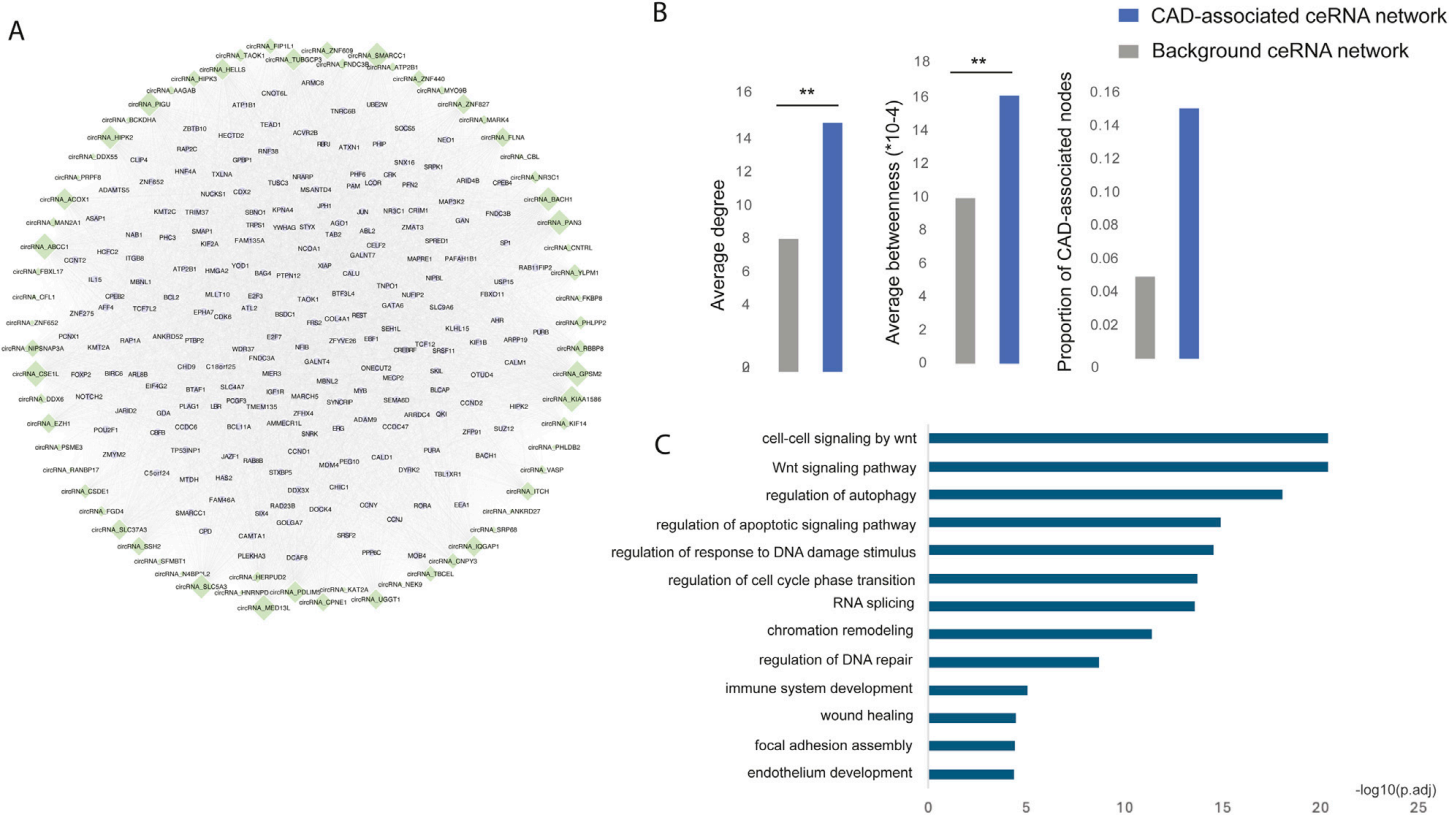
**(A)** The risk circRNA-mRNA competitive regulatory network in CAD is illustrated, with green diamonds representing circRNAs and light purple circles representing mRNAs. Node size represents network degree. **(B)** The network topological properties of CAD-associated ceRNA and background network. **(C)** Significantly enriched GO functions of the risk circRNA network.

To identify the CAD risk circRNA-mRNA competitive regulatory network, we retained circRNA-mRNA interactions involving at least one CAD GWAS or known or DE circRNAs or genes. This approach identified a total of 74,307 circRNA-mRNA interactions, involving 69 circRNAs and 8,540 genes. The top 200 genes with high network degree were shown in Figure 3A. Notably, known CAD circRNAs exhibited the highest network degrees, including circNIPSNAP3A (Wang et al., 2024), circZNF609 (Liang et al., 2020), circHIPK3 (Zhang et al., 2021), and circHERPUD2 (He et al., 2023). Additionally, circABCC1, circKIAA1586, and circGPSM2 emerged as top-ranking circRNAs in terms of network degree, potentially serving as key regulators in CAD.

Multiple topological and functional properties of the ceRNA network were analyzed against the background ceRNA network (Figure 3B). Two widely used topological properties, degree and betweenness, were calculated to investigate the important roles of disease-associated ceRNA network. Network nodes with high degree are highly connected and considered as hubs and nodes with high betweenness control the extent of information flow and are referred to as bottlenecks. We found that nodes in the disease ceRNAs had significantly higher degree and betweenness than those in the background ceRNAs. This comparison indicated that nodes in disease ceRNAs tended to be the network hubs and bottlenecks, implying important functions.

To elucidate the biological functions of the CAD risk circRNA network, we conducted GO function enrichment analysis (FDR <0.05) for genes within the network. This analysis revealed significant enrichment in biological processes associated with CAD, such as the Wnt signaling pathway (Weerackoon et al., 2021), wound healing (Li et al., 2017), endothelium development (Medina-Leyte et al., 2021) and immune system development (Feng et al., 2022) (Figure 3C).

### EC-specific circRNA regulatory network in CAD

**FIGURE 4:**
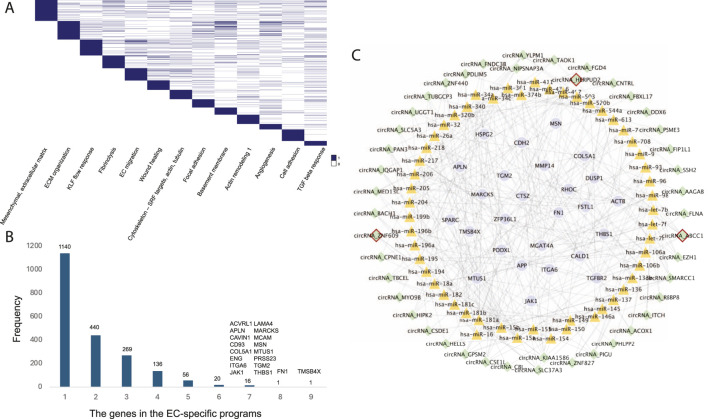
**(A)** The 13 EC-specific gene programs in CAD identified by Jesse M. Engreitz’s study. **(B)** The frequency distribution of genes across the EC-specific programs. **(C)** The EC-specific circRNA regulatory network in CAD. Nodes with red borders represent known or GWAS-associated circRNAs.

Endothelial cells (ECs) play a crucial role in the development of atherosclerosis, a leading cause of CAD. In a study by Jesse M. Engreitz (Schnitzler et al., 2024), 13 EC-specific gene regulatory programs related to CAD were identified using perturb-seq and single-cell RNA-seq techniques. Each program included 300 genes, resulting in a total of 2,019 genes across all EC-specific programs (Figure 4A). To uncover common CAD pathogenesis in ECs, we identified genes present in at least six of these programs (Figure 4B).

Further analysis revealed 44 circRNAs regulating these common target genes by mediating 53 known CAD-associated miRNAs (Figure 4C). Among these, circHERPUD2, a known CAD biomarker (He et al., 2023), was highlighted. Our findings indicate that circHERPUD2 competitively binds to hsa-miR-218, subsequently regulating the gene *MARCKS*, which is implicated in several EC-specific programs, including extracellular matrix (ECM) organization, KLF flow response, focal adhesions, basement membrane, angiogenesis, and cell adhesion.

### Connecting variants to circZNF609 and circABCC1 in EC dysfunction

**FIGURE 5:**
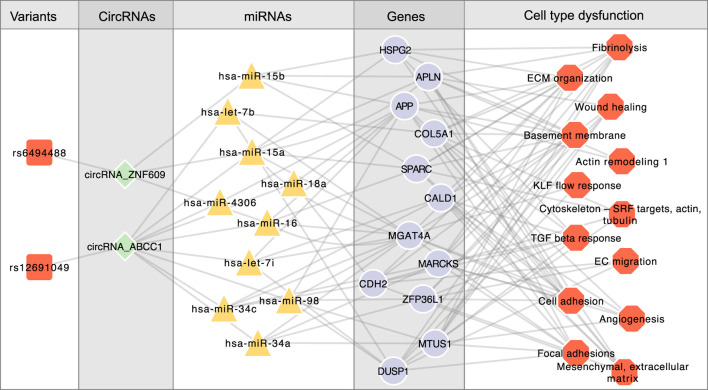
Linking CAD variants to circZNF609 and circABCC1 in EC-specific programs, illustrating the crosstalk between CAD risk variants, noncoding regulators (circRNAs and miRNAs), target genes, and the corresponding cellular contexts.

Endothelial cells (ECs) are significantly implicated in CAD heritability (Turner et al., 2022). We identified 90 circRNAs in proximity to 307 CAD-associated variants (within 500 kb). Liu et al. demonstrated that silencing circular RNA-ZNF609 ameliorates vascular endothelial dysfunction (Zhang et al., 2020a; Liu et al., 2017). In our study, we found that CAD variant rs6494488 is located on circZNF609. This circRNA competitively bind to hsa-miR-15a, hsa-miR-15b, and hsa-miR-16, thereby regulating the expression of *SPARC*, *HSPG2*, *MGAT4A*, *APLN*, and *APP*, all of which are involved in EC-specific programs such as ECM organization, cell adhesion, and basement membrane maintenance (Figure 5).

Another CAD GWAS variant, rs12691049, is associated with circABCC1, which regulates the largest number of target genes within the EC-specific circRNA regulatory network. Our findings suggest that circABCC1 promotes EC dysfunction by modulating several CAD-associated miRNAs, including hsa-miR-34a, hsa-miR-34c, hsa-let-7b, and hsa-let-7i (Figure 5). These results highlight circZNF609 and circABCC1 as potential key regulators in the pathogenesis of CAD.

We explore the crosstalk between CAD risk variants, noncoding regulators (circRNAs and miRNAs), target genes, and the corresponding cellular contexts. It highlights how variants influence noncoding RNAs like circZNF609 and circABCC1, which in turn regulate key genes involved in endothelial cell dysfunction, thereby providing insight into the molecular mechanisms underlying CAD.

## Discussion

Our study offers new insights into the molecular mechanisms underlying CAD by examining the regulatory roles of noncoding RNAs, particularly circRNAs, in ECs. By identifying and integrating CAD-associated circRNAs, miRNAs and gene sets, we constructed a comprehensive circRNA competitive regulatory network. This network illustrates the complex interplay between genetic variants and noncoding RNAs in the context of CAD.

A key finding of our study is the disruption of circZNF609 and circABCC1 by specific CAD-associated variants. These circRNAs function as miRNA sponges, modulating gene expression in endothelial cells. The dysregulation of these gene programs may contribute to CAD pathogenesis by impairing endothelial cell function and promoting atherosclerosis.

The identification of circZNF609 and circABCC1 as critical regulators in CAD underscores the significance of circRNAs in the disease process. Our findings suggest that circRNAs play a vital role in gene regulation and disease progression, adding a new layer of complexity to the genetic architecture of CAD. This underscores the need for further research into the functional implications of circRNAs in cardiovascular diseases. Integrating GWAS data with circRNA regulatory networks has proven to be an effective approach for linking genetic variants to disease mechanisms. Our study demonstrates the utility of this approach in uncovering the molecular basis of complex diseases like CAD. By identifying specific circRNAs affected by CAD-associated variants, we provide a clearer understanding of how genetic predisposition translates into disease risk at the molecular level. This knowledge could inform the development of targeted therapies aimed at modulating circRNA activity to reduce CAD risk.

However, several limitations must be addressed in future studies. While our network model offers valuable insights, it is based on bioinformatics predictions and requires experimental validation. Functional assays are needed to confirm the roles of circZNF609 and circABCC1 in endothelial cell dysfunction and CAD pathogenesis. Additionally, our study focused on a limited number of circRNAs and genetic variants. Expanding this analysis to include a broader range of circRNAs and variants could provide a more detailed understanding of the regulatory landscape in CAD. The EC-specific circRNA subnetwork identified in our study consists of differentially expressed genes that distinguish CAD patients from healthy controls, underscoring its potential utility in predicting CAD risk.

With the rapid advancement of single-cell and spatial technologies, the investigation of disease mechanisms and key molecular players, including circRNAs, has greatly progressed. Cutting-edge computational methods such as scDRS and SCAVENGE facilitate the efficient integration of disease-associated genetic variants with diverse single-cell datasets, including scRNA-seq and scATAC-seq, enabling the identification of disease regulatory circuits at unprecedented resolution. Our study underscores the promising role of circRNAs in uncovering potential therapeutic targets for CAD. Future research should prioritize the development of novel computational algorithms, particularly AI-powered approaches, to comprehensively analyze non-coding RNAs—including but not limited to circRNAs—at single-cell resolution.

In conclusion, our study highlights the critical roles of circRNAs in the genetic regulation of CAD and establishes a link between genetic variants and endothelial cell-specific gene programs. These findings enhance our understanding of the molecular mechanisms driving CAD and open new avenues for targeted therapeutic interventions.

## Data Availability

The datasets presented in this study can be found in online repositories. The names of the repository/repositories and accession number(s) can be found in the article/Supplementary Material.

## Supplementary material

SUPPLEMENTARY FIGURE S1
**(A)** Overview of CAD-associated circRNAs and CAD-associated genes **(B)**.

SUPPLEMENTARY TABLE S1 The summary of CAD-associated circRNAs, miRNAs and genes.

SUPPLEMENTARY TABLE S2 The DE circRNAs and genes in CAD.
