# Supplementary figures and images for: Comprehensive analysis of non-coding RNA-mediated endothelial cell-specific regulatory circuits in coronary artery disease risk
Source: Front Genet. 2025 Feb 21;16:1559798. doi: 10.3389/fgene.2025.1559798 (PMC11886895; doi:10.3389/fgene.2025.1559798)

A

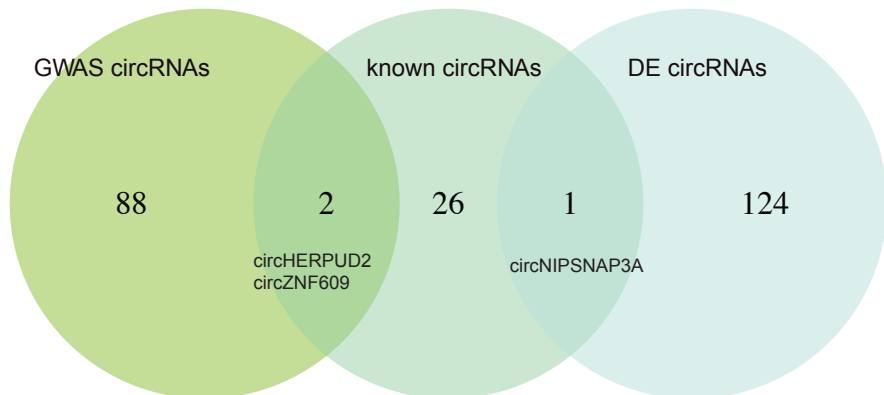

B

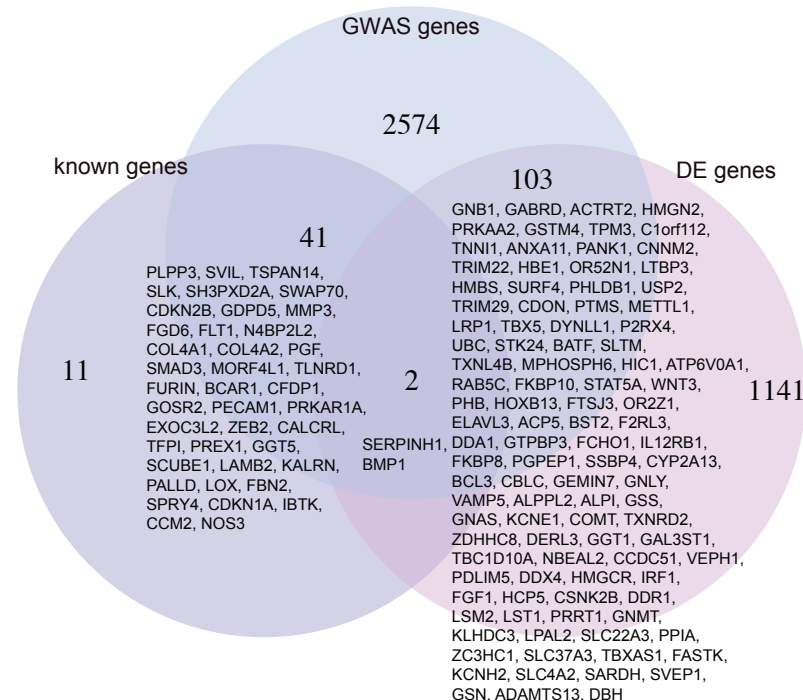

Supplement: Supplementary file 2 [file DataSheet1.pdf]
